# Clinical electives in China: trends, experiences, barriers

**DOI:** 10.1186/s12992-022-00889-3

**Published:** 2022-11-18

**Authors:** Maximilian Andreas Storz

**Affiliations:** Department of Internal Medicine II, Centre for Complementary Medicine, Faculty of Medicine, Freiburg University Hospital, University of Freiburg, Freiburg, Germany

**Keywords:** Abroad elective, International medical elective, Oversea elective, Medical education, Learning, Global health, China

## Abstract

In recent decades, China has quickly transformed itself into a modern, urban, technological and economic powerhouse. China’s medical education system is internationalizing and attracting a considerable number of foreign students seeking medical degrees and other clinical experience, such as observerships, in China. Although the majority of international students in China come from low- and middle income countries, China’s rise towards the world’s largest medical education system also offers new opportunities for stronger cooperation with European countries. Both sides maintain tight economic ties, and China’s rise is also attracting more and more medial students for short-term clinical electives from the German-speaking countries. Such clinical electives are pertinent to global health education in a globalized world, and allow students to immerse in foreign healthcare systems for a short period. Notably, reasons for (and barriers to) electives in China are largely unexplored. To address this gap, we reviewed 4 popular German elective report databases and extrapolated key characteristics of electives in China undertaken by German-speaking medical student. N *=* 40 elective testimonies were analyzed with regard to students’ elective experience, elective barriers and organizational aspects. The vast majority of students reported an elective in Shanghai (*n =* 29, 72.50%). More than 70% of students applied directly to an elective program for foreign students, whereas less than 25% applied through a bilateral exchange program. Frequently cited positive aspects of electives in China included the Chinese hospitality and the regular high-quality teaching in English for international students. Notably, almost half of student reported some kind of difficulties during their elective (*n* = 18, 45%), including language barriers (*n* = 6), administrative issues (*n* = 5) and visa problems (*n* = 2). Our data suggest that international electives in China were overall well-rated by German-speaking students. The combination of structured clinical elective programs with English supervision and the opportunity to learn more about the Chinese culture apparently attracted said students in the past 2 decades.

## Background

In the last decades, China has quickly transformed itself into a modern, urban, technological and economic powerhouse [1]. The Chinese health industry and healthcare system have been showing steady signs of improvements [1, 2], and China’s engagement with global health governance is continuously evolving [3].

With the 2019 government-set strategic goal of “Healthy China”, the country increased its medical education system’s training capacities and aimed to establish a high-quality health service system [4]. China continuously sought to explore the reform of training mode in medical education, integrated new and innovative teaching contents, optimized educational objectives, and gradually moved from a knowledge-based to a competency-based model [5, 6].

The country’s clinical medical education system is also internationalizing [7], particularly in its commitment to international education standards [8]. On par with global standards, China has also launched several ambitious reforms to cultivate physicians with the professional, clinical competencies and humanistic spirit, which are essential for the quality of clinical services demanded by the Chinese people [9].

China nowadays has the world’s largest medical education system [9], and is attracting a considerable number of foreign students seeking medical degrees and other clinical experience, including observerships and clinical research [10].

Although the majority of international students come from low- and middle income countries in Asia and Africa [10], China’s rise also offers new opportunities for greater cooperation with European countries [11]. Both sides maintain tight economic ties [11], and China’s rise towards the world’s largest medical education system is also attracting more and more students for short-term medical electives from the German-speaking countries [12].

International medical electives (IMEs) are pertinent to global health education and allow students to immerse in foreign healthcare system for a short period [13]. IMEs have been associated with various educational benefits, potentially increasing students’ medical, cultural and linguistic competences [14]. Abroad electives are traditionally popular in Germany, and there are several anonymous open-access databases cataloguing elective testimonies written by students that went abroad for an elective [15].

For this brief report, we reviewed 4 popular German elective report databases (including Famulatur-Ranking, PJ-Ranking and ViaMedici [16–19]) and extrapolated key characteristics of IMEs in China undertaken by German-speaking medical students. Based on these reports, we sought to gain additional insights into motivations for and barriers to clinical elective experience in China.

## Methods

The methods have been discussed elsewhere in great detail [12]. In brief, we reviewed 4 open-access databases for elective testimonies cataloguing reports written by German-speaking medical students for other students to share their elective experience [16–19]. Such databases are freely accessible, anonymous, and offer unique retrospective analyses opportunities. While likely not representative of the entire German medical student body [20], they allow for new insights into drivers for and barriers to IMEs in China.

For this brief report, we captured data by convenience sampling and included all reports with the geographical tag “China” published within the last 2 decades (2002–2022). Reports were included irrespective of their length. Both reports in German and English language were considered. Key characteristics including the precise elective destination, the elective year, the elective discipline and duration, and the elective rating were extrapolated to a Microsoft Excel sheet, wherein descriptive analysis of said data was performed. The aforementioned elective ratings referred to a subjective “overall elective experience”, and were not based on a clear rubric to guide students in their rating process.

## IMEs in China

### Elective destinations and disciplines

**Fig. 1:**
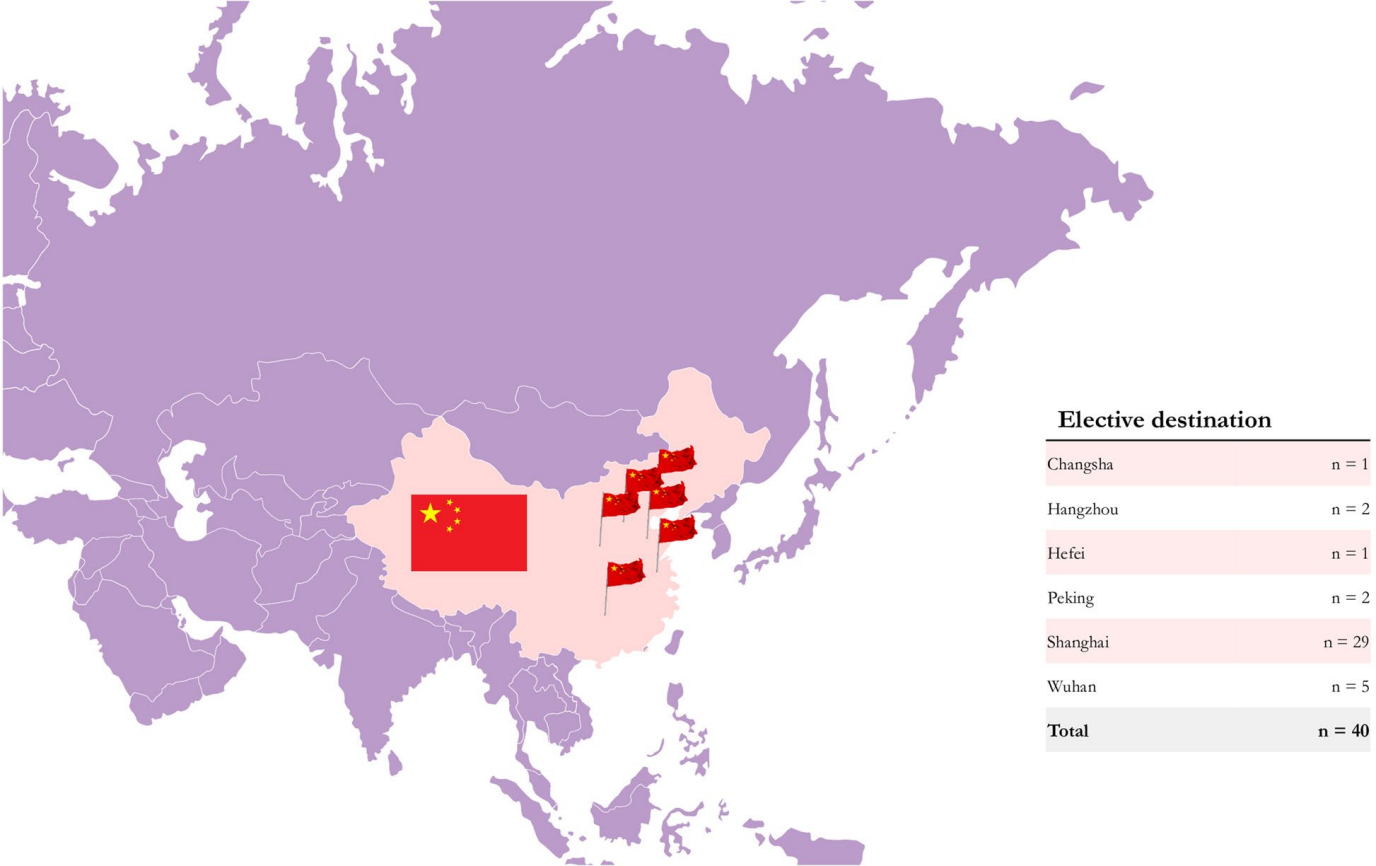
IMEs in China: Elective destinations. Modified and edited from Servier Medical Art database by Servier (Creative Commons 3.0)

We extracted a total of *n =* 40 elective testimonies from all 4 databases uploaded in the last 2 decades. The vast majority of students reported an elective in the city of Shanghai (*n =* 29, 72.50%), followed by Wuhan (*n =* 5, 12.5%). Figure 1 displays the other elective destinations reported in our sample.

Table 1 summarizes the chosen elective disciplines. With 50% of total elective testimonies, general surgery was by far the most popular discipline in our sample, followed by internal medicine (12.5%). Surgical disciplines accounted for 65% of total electives.

**Table 1:** IMEs in China: An overview of reported elective disciplines

Elective discipline	*Number of reports*
Anesthesiology	*n* = 1
Cardiology	*n* = 1
Dermatology	*n* = 1
General Medicine	*n* = 2
General Surgery	*n* = 20
Gyncology & Obstetrics	*n* = 2
Internal Medicine	*n* = 5
Neurology	*n* = 1
Neurosurgery	*n* = 1
Otorhinolaryngology	*n* = 1
Plastic Surgery	*n* = 2
Pneumology	*n* = 1
Traditional Chinese Medicine	*n* = 1
Visceral Surgery	*n* = 1
**Total**	***n*** **= 40**

### Application details

The vast majority of elective testimonies (*n =* 37, 92.5%) included organizational details on how the elective placement was organized and secured. 20% of students applied through a bilateral exchange program and indicated that their university had a memorandum of understanding with a Chinese university. The remaining 72.5% of students applied directly to a medical student elective program for foreign students (mostly at Shanghai East Hospital), contacting the respective hospitals on their own initiative.

The maximum number of testimonies per year was published in 2019 (*n =* 7), followed by a sharp decline in 2020 (*n =* 1).

### Motivations

Only a few students (*n =* 7, 17.5%) shared information about their motivations for an abroad elective in China. Gaining new cultural insights (*n =* 7, 100%) and learning in a different healthcare system were the most frequently cited reasons (*n =* 6, 85.71%), followed by the desire to travel and to holiday (*n =* 2, 28.57%). One student felt attracted “by the media controversies about the People’s Republic of China”, and endeavored to experience the life in China first-hand for a short period of time. Another student reported having Chinese roots, and aimed to learn more about the origin of her grandparents.

### Students’ experience and elective ratings

A large number of students (*n =* 38, 95%) shared details about their positive elective experiences. Many reports highlighted the Chinese hospitality (*n =* 33) and a comparable number of students (*n =* 29) expressed their happiness about receiving high-quality teaching in English language. *N =* 33 students valued the various opportunities to travel and enjoyed the permitted free time to explore the Chinese culture outside of the hospital. Occasionally, students highlighted the lower living expense (*n =* 2), the quality of the Chinese hospital kitchens (*n =* 5) and the high standard of technical devices in operating theatres (*n =* 3).

Notably, several visiting students reported difficulties during their China electives (*n =* 18, 45%). Language barriers were frequently cited (*n =* 16), although it was not always ascertainable whether this referred to visiting students’ Chinese skills or the English language skills of the respective hosts. The latter might be more likely, given that some Chinese hospitals advertise for their offered elective opportunities with “lectures and bedside teaching in English”.

A minority of students reported not having received enough teaching (*n =* 8). Administrative problems with the hospital administration (*n =* 5) and visa problems (*n =* 2) were occasionally cited. *N =* 4 students had difficulties to find a suitable accommodation in vicinity to the hospital during their elective.

Despite these barriers, students rated their China electives in a positive way (based on *n =* 33 available ratings). *N =* 20 students had an excellent experience, rating their overall elective experience with an “A” (German grade “1”). *N =* 8 students rated their elective with a “B” (German grade “2”). Only *n =* 4 students were dissatisfied, and rated their elective with a “D” or worse.

## Discussion

Using convenience sampling data from 4 open-access elective databases, we sought to gain additional insights into motivations for and barriers to clinical elective experience in China. The analyzed elective testimonies and the very good (yet subjective) student ratings suggest that IMEs in China are potentially attractive for students from the German-speaking countries. The combination of structured clinical elective programs with English supervision, and the opportunity to learn more about the Chinese culture attracted many students in this small convenience sample from the past 2 decades. Yet, the reported challenges encountered by the elective students may also provide insights for improvement.

Several authors argued that IMEs foster important medical competences, including professional identity formation, interest in humanitarian efforts and volunteerism, and value-based communication as well as personal growth [21–23]. This may particularly the case with regard to structured electives, which are arranged through reciprocal faculty partnerships or philanthropic sponsorships, and which are characterized by continuous student supervision during the entire elective [24, 25].

Yet, most electives are still largely unstructured, and oftentimes student-driven [26]. This implies that students determine the elective destination on their own, and apply individually on a case-by-case base for an elective rotation at their desired host institution(s). Unstructured electives are thus often characterized by a lack of guidance, peer support and monitoring at all elective stages (including application, preparation, conduct and de-briefing). Such electives have been repeatedly criticized as potentially harmful and detrimental for trainees, sending institutions, and – above all - the host community [26, 27]. Such electives potentially contribute to the exploitation of vulnerable healthcare systems, and may result in ethical dilemmas [15].

While a detailed discussion of that particular topic is beyond the scope of this brief report, our data suggest that only 1/5 of students applied through a bilateral exchange program. The large majority of elective experience in this sample was self-organized. Whether this type of elective experience served as a rigorous channel of medical learning was unfortunately not ascertainable from the available data. We thus clearly acknowledge that our descriptive analysis is not based on actual measurements of learning competencies but based on students’ self-reports and self-assessments.

Whether for example the frequently reported “high-quality teaching in English for international students” actually translated into measurable improvements in medical skills and knowledge was not ascertainable from our data, and would also be beyond the scope of this analysis. Prospective interventions studies would be required for such questions, yet most elective research is based on retrospective cross-sectional analyses [28, 29].

However, apart from medical knowledge, we also argue that IMEs have additional assets. They may help to improve cross-cultural conversation [21], and potentially help to facilitate international exchange and dialogue in a world of increasing global geopolitical tensions and conflicts. Based on our tertiary experience, IMEs may constitute a platform of commonalities and intercultural exchange [30], facilitating cooperation in a key area of common interest: the promotion of global environmental and human health. Then again, one must also question whether it is indicated to offer electives to individuals looking for “travel and holiday” (as reported by some students in our sample) in a time when health workforce resources are so thinly stretched.

Regrettably, our data does not allow for an answer to these questions but suggests that students generally enjoyed their elective experience. The fact that most students in our sample rated their electives exceptionally well (grade: A), despite the non-negligible existence of language barriers (Chinese is not a common language taught in Germany), is an indirect indicator that IMEs serve a purpose far beyond simply exchanging medical knowledge and ideas.

International medical electives, as part of structured global surgery academic partnerships between institutions in high-income countries and low-middle income countries, may also play a pivotal role in developing surgical workforce capacity [31–33]. The latter is of utmost importance, given that nearly 5 billion of the world’s growing population lacks access to safe, accessible and equitable surgical care. In our sample, general surgery was the most frequently chosen elective discipline, and surgical disciplines accounted for 65% of total electives. Said factor must be kept in mind when discussing about the potential pros and cons of international electives, and is a noticeable feature of our sample.

While our report does not resolve the ongoing debate on the value (and pros and cons) of IMEs, we may contribute important data that facilitate the understanding of elective experience in China. Although largely from pre-pandemic times, our data may allow for important first insights in motivations for (and barriers to) electives in China. Data from the last 2 years would be most desirable, but is largely unavailable due to COVID-19 related travel restrictions and closure of international elective programs [13, 20].

The fact that the majority of elective experience in this sample was largely Shanghai-centered could be interpreted in a way that students mainly sought elective experience in one of China’s most dynamic and developed metropoles, which is a mix of East and West. Electives in the Western parts of China were rarely reported. Then again, Shanghai concentrates hospitals that offer regular teaching in English for foreign students during their elective – an important feature and magnet for all students who are unfamiliar with the Chinese language but seek to visit the country anyway [34].

### Strengths and limitations

This brief report has strengths and limitations to discuss. The reservation must be made, that from a quantitative perspective the number of identified elective reports was only modest (*n =* 40). Notably, writing elective testimonies is not mandatory, and students usually receive no credit for this work [15]. Thus, it is likely that the actual number of students going to China for an abroad elective was much higher. Our sample is a convenience sample and may not be representative of the German medical student body as a whole.

As reported earlier, student testimonies are often subject to recall bias and may not replace face-to-face interviews [15]. In light of the COVID-19 pandemic and the COVID-related decline in elective activities, however, they serve a valuable purpose, are easily accessible and allow for additional insights into abroad elective trends.

## Conclusion

While limited by its sample size, the present report gives important insights into motivations for and barriers to clinical elective experience in China. The emphasized elective features and experience may be useful for elective organizers and host institutions to modify and improve their elective programs. Ultimately the extrapolated and analyzed elective testimonies give hope as they could be carefully interpreted as microdocuments of cross-cultural exchange and dialogue between China and Europe in a world of increasing economical tensions and environmental problems threating human life on this planet.

## Data Availability

All data associated with this paper will be made available upon reasonable request.

## Abbreviation

IME: International Medical Elective
